# Reverse perfusion pattern in myocardial spect with 99mTc-SestaMIBI

**Published:** 2013-09-25

**Authors:** O Schillaci, M Tavolozza, D Di Biagio, A Lacanfora, A Chiaravalloti, E Palombo, R Catalano, G Simonetti

**Affiliations:** “Department of Biopathology and Diagnostic Imaging", “Tor Vergata" University of Rome, Rome, Italy

**Keywords:** 99mTc-Se staMIBI, myocardial SPECT, reverse distribution, reverse perfusion

## Abstract

**Rationale.** The aim of our study was to investigate the myocardial perfusion deficit in rest images as compared to stress images in myocardial scintigraphy (MS).

** Objectives. **The aim of this study is to investigate the reverse perfusion (RP) pattern in MS.

** Methods and Results. **263 patients were enrolled in the study (72 females and 191 males; mean age 65.7 ± 9.5 years old). Mean body mass index (BMI) was of 27.6 ± 3.8 Kg/m2. 115 patients were positive for a previous history of myocardial infarction (MI). 142 patients reported a revascularization treatment (percutaneous transluminal coronary angioplasty, PTCA, cardiac stent placement, coronary artery bypass grafting, CABG). All the patients underwent MS following standard single day Stress/Rest protocol.

In our series, 27 patients presented a RP pattern. We did not find statistically significant differences when considering age (p = 0.7988), sex (p = 0.0657), BMI (p = 0.8611), diabetes (p = 0.8259), dyslipidemia (p = 0.1464) or smoking status (p = 0.6829) in RP patients vs. non-RP patients. A history of MI is related to a RP pattern (p < 0.0001). A history of previous revascularization was not related with RP (p = 0.6868).

**Discussion.** The result of our study suggested that RP is probably related to artifacts of various origins. Further studies are necessary especially in microvascular dysfunction or a long history of disease.

**Abbreviations: **MS: myocardial scintigraphy, RR: reverse redistribution, RP: reverse perfusion, MI: myocardial infarction, CAD: coronary artery disease, CABG: coronary artery bypass grafting; PTCA: percutaneous transluminal coronary angioplasty; PCI: percutaneous coronary intervention, SPECT: single-photon emission computed tomogram.

## Introduction

In myocardial scintigraphy (MS), the term “reverse redistribution" (RR) consists in the appearance or in the worsening of a perfusion defect, generally on Thallium-201 (201Tl), in the rest of the images in comparison with the stress images [**1,2**]. According to the well-known feature of 201Tl this isotope is actively captured by the myocardial cells by Na-K pump and then redistributes itself proportionally to the dynamic equilibrium with the ion from the extracellular pool [**3**]. 

 Both Technetium-99m-methoxyisobutyl-isonitrile [99mTc-SestaMIBI (MIBI)] and 99mTc-Tetrofosmin are monocation soluble compounds and are used as myocardial perfusion tracers [**4,5**]; their retention requires cellular viability, in particular cell membrane integrity and preserved mitochondrial function. These compounds are characterized by a persistent retention within mitochondria [**3,6,7**], reflecting not only flow, but also myocardial viability [**8**].

 Several studies show that, in normal myocardial muscle, 99mTc-MIBI, does not significantly re-distribute in the cardiac muscle [**9,10**]. Therefore, the delayed acquisitions still show myocardial perfusion at the time of injection [**11**].

 In 1979, Tanasescu et al. first described the RR scintigraphic appearance and until now, there has not been a unifying theory that explains this pattern [**12,16**]. The presence of stunned myocardium (that is not able to retain Tetrofosmin as long as normal cells) or a fourth mechanism has been proposed for a possible explanation of a RR pattern [**17**].

 Many authors compared early and delayed images (1 hour and generally three hours after the injection) to study the RR of 99mTc-MIBI in the same modality of 201Tl [**6,8,21**]. In patients with coronary artery disease (CAD), a more rapid wash-out of 99mTc-MIBI from territories presenting a mixture of hibernating myocardium with necrosis or “stunned" has been reported [**17,21**].

 When considering MS with 99mTc-Tetrofosmin, the presence of one or more areas of reduced tracer uptake in comparison with the stress images is actually called “reverse perfusion" (RP) and, considering the dynamic of this radiolabeled compound, the phenomenon has been explained with the presence of myocardial perfusion abnormalities, albeit at the microvascular level [**7**]. 

 The aim of our study was to investigate the rate and features of RP in a population with myocardial infarction (MI) or with suspicion of CAD.

## Materials and methods

**Patients** We examined 263 consecutive patients (72 females and 191 males; mean age 65.7 ± 9.5 years old) that underwent MS with 99mTc-MIBI according to the standard guidelines [**23**]. A general overview of the whole population is shown in Table I.

**Table 1:** Baseline characteristics of the two populations of ambulatory patients who underwent technetium-99m single photon emission computed tomography between November 2010 and March 2011.

Characteristic	RP group (n 27)	non-RP group (n 236)
Age (years)	65.3 ± 8.7	65.8 ± 9.5
Male sex - number (%)	24 (88.9)	167 (70.8)
BMI	27.8 ± 2.23	27.6 ± 3.98
Diabetes - number (%)	7 (25.9)	68 (28.8)
Dyslipidemia - number (%)	13 (48.1)	149 (63.1)
Smoking - number (%)	17 (63.0)	136 (57.6)
History of - number (%)		
- Myocardial infarction	20 (74.1); (17.4)†	95 (40.3); (82.6)†
- CABG*	3 (11.1); (15.8)‡	36 (15.3); (29.3)‡
- PTCA* - Stent (PCI*)	12 (44.4); (63.2)‡	83 (35.2); (67.5)‡
- Thrombolysis	4 (14.8); (21.1)‡	10 (4.2); (8.1)‡
- CABG *+ PCI*	0	6 (3); (5)‡
Exercise SPECT* -number (%)	24 (88.9)	202 (85.6)
Dipyridamole SPECT* - number (%)	3 (11.1)	34 (14.4)
* CABG = coronary artery bypass grafting; PTCA = percutaneous transluminal coronary angioplasty; PCI = percutaneous coronary intervention; SPECT = single-photon emission computed tomography. † percentage calculated on the total of patients with myocardial infarction. ‡ percentage calculated on the total of patients with a history of revascularization.		

 The patients fasted overnight and all cardiovascular drugs were withdrawn 3 days prior to the study [**24**].

 Most of our patients were asked to exercise on a treadmill, using a standard Bruce protocol [**25,26**] (n 227, 86%); the exercise was terminated if one or more of the following symptoms were present: fatigue, dyspnoea, angina pectoris, dizziness, ST-segment depression, hypotension and arrhythmias [**25,26**]. Thirty-seven patients (14%) with left bundle branch block or who were unable to exercise were tested by using intravenous dipyridamole (0.56 mg/kg in 4 minutes) according to standard guidelines [**27**].

**Scintigraphy**

 We adopted Stress/Rest protocol with 300 MBq 99mTc-MIBI at peak exercise during bicycle ergometry and 900 MBq at rest following standard guidelines [**24**].

 Single Photon Emission Computed Tomography (SPECT) was performed on a GE Discovery ST equipped with an LEHR collimator. Thirty-two images of 25 sec per frame at stress and 20 sec per frame at rest (matrix 64 x 64, zoom 1.33) were acquired using the “step and shoot" technique (90 g/head). The images were acquired along a circular orbit; range was of 180° from 45° RAO to 45° LPO. Energy discrimination was provided by a 20% window centered over the 140 KeV Photon peak of 99mTc. Transverse images were reconstructed by the filtered backprojection method, with a Butterworth filter (order 10; cut-off 4.0) for processing and a Ramp filter for backprojection [**24**].

**Imaging interpretation**

**Fig. 1:**
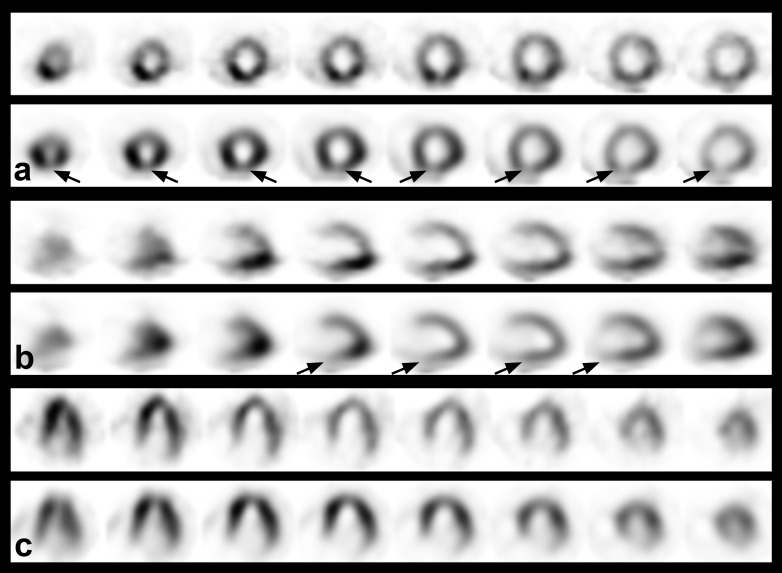
Stress and Rest 99mTc-MIBI single-photon emission computed tomography images aligned in standard short axis (a), vertical long axis (b), and horizontal long axis (c) format revealing an area with reduced 99mTc-MIBI uptake at rest imaging in the lower wall segment (reverse perfusion pattern).

 The presence of a RP pattern was defined as a worse or new defect on the rest image compared with the stress image, on at least two consecutive slices as previously performed in other studies at visual inspection (**Fig. 1**) [**28**]. SPECT images were analyzed by two experienced observers (O.S. and M.T.) and then classified based on the presence (RP patients) or absence (non-RP patients) of a RP pattern. 

**Statistical analysis**

 Continuous variables were compared by a student’s t test and the differences in proportion (categorical variables) were examined by Fisher’s exact test or the chi square test.

 A p value of ≤ 0.05 was considered significant.

## Results

The SPECT images of 27 of the 263 patients examined were consistent with the RP pattern (10.3%).

 Baseline characteristics of RP patients and non-RP patients are shown in Table I. 

 We did not find statistically significant differences in gender when comparing the two groups (p = 0.0657).

 We did not find differences in BMI when comparing RP patients and non-RP patients (RP = 27.8 ± 2.23; non-RP = 27.6 ± 3.98, p = 0.8611), in age (RP = 65.3 ± 1.7; non-RP = 65.8 ± 0.6, p = 0.7988), presence or absences of diabetes (p = 0.8259), dyslipidemia (p = 0.1464) or smoking status (p = 0.6829).

 The comparison between the rate of patients who have undergone revascularization in RP group (coronary artery bypass grafting, CABG, percutaneous transluminal coronary angioplasty, PTCA, and cardiac stent placement; n = 15, 55.6%) and in the non-RP group (n 119, 50.4%) did not show a statistically significant difference (p = 0.6868); We found a significant difference in the number of subjects who underwent thrombolysis (n = 4, 14.8%, p = 0.0432) in our series.

 A history of MI was significantly related to a RP pattern (RP n = 20, 74.1%; non-RP n = 95, 40.3%, p < 0.0001).

 The time elapsed after MI and/or revascularization procedure was over 2 years (Tables II and III).

**Table 2:** Time IMA-SPECT

≤ 3 months	1	5.0%
3 < x ≤ 6 months	1	5.0%
6 < x ≤ 12 months	3	15.0%
1 < x ≤ 2 years	2	10.0%
> 2 years	13	65.0%
	20	100.0%

**Table 3:** Time Revascularization-SPECT

≤ 3 months	1	5.3%
3 < x ≤ 6 months	2	10.5%
6 < x ≤ 12 months	3	15.8%
1 < x ≤ 2 years	1	5.3%
> 2 years	12	63.2%
	19	100.0%

 We did not find any significant difference between RP and non-RP patients when considering the time elapsed from the day of the MI and/or revascularization to the day of MS (p = 0.0625 and p = 0.4648 respectively).

 The sites of MI and revascularization coincide in 12 and 14 patients respectively (60% and 73,7% of the patients with MI or revascularization).

 The site of MI and revascularization is not related to RP (p = 0.7674 and p = 0.426).

 RP pattern occurred in the anterior wall and/or sep¬tum, in 8 patients while the inferior wall was the most frequent site of RP (18 patients). Only one patient (male, BMI 26.4) showed an RP deficit in the lateral wall.

 Five patients with no history of MI and/or revascularization have shown an RP pattern.

## Discussion

The main finding of our study is that RP pattern is related to a previous history of MI.

 RR in 201Tl MS has already been studied in the past with controversial hypotheses about its etiology [**1,2**]. According to a well-known feature of the 201Tl (that is taken up by active myocardial cells by using the Na-K pump, redistributing itself proportionally to the dynamic equilibrium with the ion from the extracellular pool) the in-vivo study of the myocardial distribution of this compound allowed the differentiation between the necrotic myocardium and the ischemic tissue, that is able to accumulate radioelement, although with a slower kinetic [**3**].

 Therefore, the RR of 201Tl was initially attributed to an ischemic phenomenon in patients with multi vessels CAD; being detected in the area with the most stenotic vessels that lead to a slower accumulation of this compound [**29**]. Nevertheless, other authors described this phenomenon in territories supplied by a vessel with recent thrombolysis and has been considered as an index of successful revascularization [**30**].

 The phenomenon of RR has also been observed with technetium-labeled compounds, using the same acquisition protocol of 201Tl (consisting in early, 30', and late, 3 hours, acquisition) [**6,18,21**] and also by means of standard MS protocols [**7,31,32**].

 Although technetium radiolabeled compounds do not exhibit the phenomenon of redistribution as monocations soluble compounds with persistent retention within the mitochondria, it has been shown that MIBI and Tetrofosmin present a rapid wash-out from territories with a mixture of hibernating necrotic or stunned myocardium [**17,22**].

 The RP has also been shown when using “single day" or “double day" MS protocols [**7,31**].

 Araujo et al. postulated that the RP is due to an artifact related to attenuation phenomena, being more frequent in subjects with higher BMI (i.e. RP pattern in the inferior wall in obese men, and anterior RP pattern in women with abundant breast) [**33**]. In this study, a Rest/Stress protocol (characterized by low radioactivity at rest) has been used in all patients [**33**]. 

 The results of our study are in partial disagreement with those of the previously mentioned reports. In fact, we did not find any correlation between BMI and the frequency of RP; furthermore, the percentage of females in the RP group is less than that of the non-RP group (11.1% vs. 29.2%) even if this last data did not reach a statistical significance in our study.

 A possible confirmation of our findings can be sought in studies investigating RP with a stress/rest MS protocol. In particular being the maximum radioactivity dose administered at rest, there is probability of attenuation artifact. 

 According to the previously published literature, the lateral wall is hardly the site of artifacts, being the attenuation deficit affects more frequently observed in the anterior or the inferior wall of the left ventricle [**34**]. Interestingly, one of our patients with a normal BMI and a history of previous MI in the lateral wall presented a RP pattern in this site contradicting the hypothesis that the RP pattern could be exclusively due to artifacts [**32**]. This assumption is at the time speculative, since the present study is limited by the small population examined and, in particular, only 3 of 263 patients had a MI on the lateral wall (2.6% of 115 patients with a history of MI), being a history of MI significantly related to RP in our study.

 Although the two groups of patients did not show any significant relationship when considering the time elapsed from the day of MI and/or revascularization and MS (p = 0.0625 and p = 0.4648 respectively), we did not find a significant correlation between RP and the presence of hibernating and/or stunned myocardium. A possible explanation can be sought in a previous history of MI. In fact, most of the patients (15 of 20 with a history of stroke [75%] and 13 of 19 with a history of revascularization [68.5%]) had a history of MI and/or revascularization dating back more than one year before the MS.

 It can be speculated that, being an RP pattern most frequently met in patients with a heart disease for a long time, a possible compensatory remodeling could play a role in this phenomenon. 

 The absence of a significant difference between the two groups in diabetes, dyslipidemia, and smoking, rules out the possibility of coronary microvascular dysfunction [**35,36**].

 In our series, a strong relationship between the RP pattern and a history of MI (p < 0.0001); no significant relationships have been found with a history of revascularization (CABG, PTCA, and cardiac stent placement; p = 0.6868). These last findings underline the possible role of a reversible or irreversible damage respectively, being the myocardial perfusion affected by compensatory mechanisms [**37**].

 In our study, there is a weak relationship between the RP pattern and a history of thrombolysis. In fact, only four of 27 patients with RP (14.8%) presented a RP pattern (p = 0.0432). This finding is probably due to the small number of patients examined. Moreover, we observed a high coincidence of RP pattern in the site of MI (60%) or revascularization (73.7%) Further studies performed on a larger pool of patients are necessary in this direction in order to confirm these findings.

 In conclusion, the preliminary data of our study suggest that the RP is a phenomenon related to ischemia but other causes can also be involved. The combination of a different distribution of coronary flow in myocardial tissue studied at rest and after the stimulation is probably due to a better perfusion in the district under stress.

**Disclosures**

 The Authors declare that they have nothing to disclose.
